# Jan Svoboda (1934–2017): sixty years with retroviruses

**DOI:** 10.1186/s12977-017-0357-2

**Published:** 2017-05-22

**Authors:** Jiří Hejnar

**Affiliations:** Institute of Molecular Genetics, Czech Academy of Sciences, Vídeňská 1083, 14220 Prague 4, Czechia

Jan Svoboda, renowned virologist and geneticist, passed away peacefully after a short disease on March 13, 2017. He was 82. With his passing, the scientific community lost a leader of retrovirology, whose pioneering work was accompanied by contagious passion for science, dignity, and encouraging charm. He was celebrated not only for his scientific achievements, but also for inspiring a generation of younger scientists and his for public activity in favor of the Czech science. Jan spent most of his career at the Institute of Molecular Genetics, Czech Academy of Sciences. He also served as Director of the Institute from 1991 to 1999.

Jan’s long and colorful life in science was tightly interconnected with the modern history of Czechoslovakia and Czech Republic, as he engagingly described in his commemorative paper (Svoboda J.: Foundations in Cancer Research. The Turns of Life and Science. Advances in Cancer Research 2008). He was born in Prague, but grew up in a small village, Dobré Pole (Good Field), during the World War II. In a middle class family, he was brought up in the spirit of democracy and awe of education. His personality was formed particularly by the enthusiasm and liberalism in the first three years after liberation of Czechoslovakia in 1945, which led to troubles in the later period of repressions after the communist coup d’état in 1948.

Jan studied biology at the Faculty of Science, Charles University in 1952–1957, and already in these years he started experiments with transforming the cells by Rous sarcoma virus (RSV). Jan’s life-long relish for cell culture dates from that time. I keep in mind that even in his later years, Jan did not regret time and maintained cells in the culture by his own hands (and never far from his pipe and sweet cherry tobacco). During his studies, he became a volunteer in the laboratory of Milan Hašek, excellent immunologist and proponent of immune tolerance. The environment in the Hašek laboratory was very friendly, open, and stimulating, also thanks to frequent contacts with foreign visitors. Jan remembered the discussions with Conrad Waddington, which provided him with many arguments against Lysenkoism, the bizarre alternative to Mendelian genetics politically favored in the Eastern Block at that time. Jan started to publish his results on RSV in immune tolerance together with Milan Hašek in 1953. Nevertheless, he still thought about the rapid cell transformation and tumor induction by RSV. His belief in the extraordinary character of RSV replication strengthened after meeting Lev Zilber, George Svet-Moldavsky and Fyodor Kisseljov in Moscow in 1957.

**Fig. 1:**
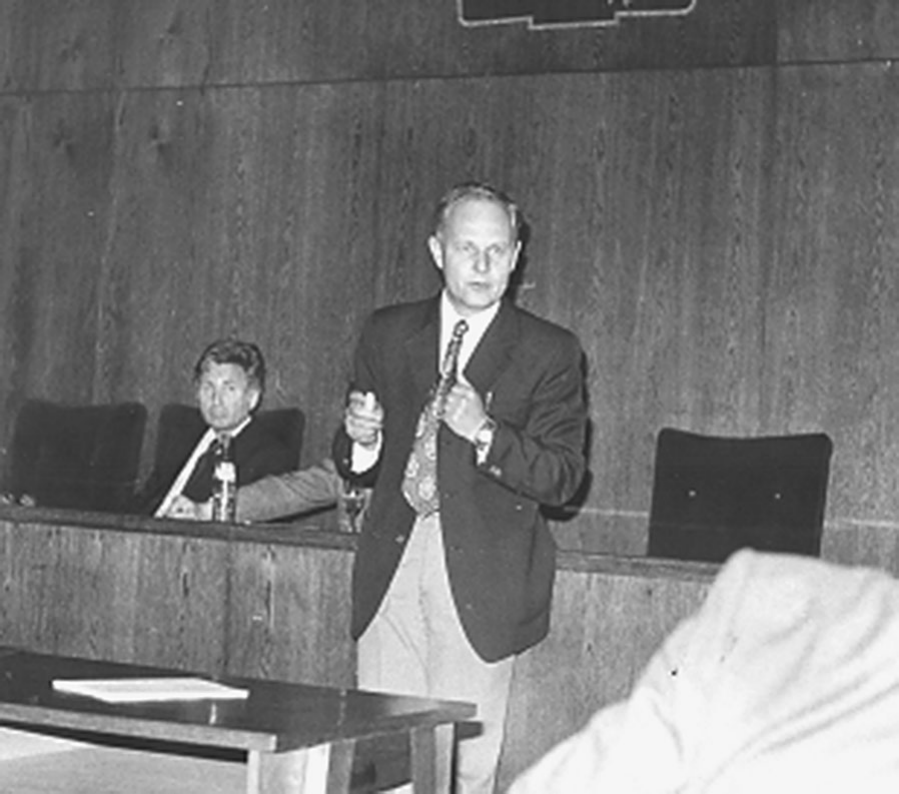
Jan Svoboda lectures at the Slovak Days of Oncology in Bratislava in 1964

Jan’s key achievement came from the infection and transformation by RSV of mammalian hosts, laboratory rats, mice and hamsters. The non-permissive environment in mammalian cells did not support RSV replication, but the virus could be rescued after injection of minced tumor cells into chickens or, later, by in vitro induced fusion of transformed cells with permissive chicken cells. The elusive persistence of latent RSV, so called virogeny, was explained by the provirus hypothesis. For detailed analysis of virogeny in XC rat cells, Jan attracted Slovak colleagues Dušan Šimkovič and others for collaboration (Fig. 1). He also corresponded with Howard M. Temin. In 1963, Jan attended the International Avian Tumor Virus Conference in Durham, NC, and met Harry Rubin, Howard Temin, Peter Vogt, Hidesaburo Hanfusa and others. Since that time, Jan remained in close contact with Western retrovirology, and his XC cells became a frequent tool in many laboratories.

As a pioneer and deep thinker, Jan Svoboda put much thought and work into elaborating multiple virogenic models and getting a versatile support for the proviral hypothesis in the late 1960s. Nevertheless, he remained alone with Howard Temin in defending this hypothesis until the biochemical mechanisms of RNA to DNA transcription and the respective enzyme, reverse transcriptase, was discovered in 1970. In his Nobel lecture, Howard Temin fairly mentioned Jan’s credit, saying that “Svoboda et al. from studies of RSV-infected rat cells independently postulated the existence of a provirus in RSV-infected cells.” Even having the enzyme capable of RNA to DNA transcription, the formal proof of proviral RSV form, virus rescue by transfection of DNA from virogenic cells remained to be done, and Jan established collaboration with Miroslav Hill on transfection of XC DNA.

The end of that fruitful period was heavily scarred by the Soviet occupation of Czechoslovakia in 1968. The critical transfection experiments with XC DNA could not be performed in Prague and later on, the experimental work became more and more difficult behind the iron curtain because of people emigrating, scarcity of materials, and personal oppression from the side of the “normalization” regime. Despite the dire situation, Jan Svoboda did not resign, reestablished his group and contributed substantially in the discovery of the *src* oncogene. Again in mammalian cells, he and his peers described cryptovirogenic proviruses containing just a small but transforming part of the RSV genome. Rescue of such proviruses required not only fusion with chicken cells, but also preinfection with a replication-competent virus, so called helper virus. Importantly, some cryptovirogenic and therefore replication-defective proviruses still kept their transformation competence, indicating that cell transformation is ensured by an independent genetic entity. Collectively, these findings contributed to the definition of the *src* oncogene and to the concept of retroviral vectors. Of remarkable consequence was the H-19 provirus, which arose by integration of the spliced v-*src* mRNA transcript and defined v-*src* as a minimal autonomous transforming RSV unit.

In the 1980s, the power of the communist regime began to rust and the political repressions died down. Jan Svoboda was allowed to travel abroad again and spent two years as visiting professor at the University of Missouri in Columbia, MO. He experimentally worked on projects brought from Prague and I remember that he directed our laboratory by frequent letters with detailed instructions to each of us. His return visits to Prague used to be marvelous: he brought dry-ice boxes full of enzymes, kits and fine chemicals, which were not available in our country at that time. These materials helped us to pursue at least some projects, but we perceived the deep technological gap between Czechoslovakia and the USA and the need to concentrate on topics emanating from Jan’s traditional models. The retrovirology scene rapidly changed after the discovery of HTLV and HIV. Jan was in contact with Jean-Claude Cherman in Paris and Mikulas Popovic at NIH who was his former Slovak collaborator, and thought about studying the cross-species transmission of HIV-1. He, however, realized that such experiments could not be done at our Institute. Instead of that, he launched our epigenetic studies based on the frequent reversion of the transformed phenotype in the H-19 cell line. In collaboration with John Wyke in Glasgow, it turned out that transcriptional silencing of the H-19 provirus accompanied by DNA methylation was behind this phenomenon, and this observation inspired our later contributions to the epigenetic characterization of HIV latency.

On several occasions, Jan told me “stick to your guns”, meaning that it pays off to pursue and cultivate one’s own topics with the help of original models and approaches. His model was RSV and other avian sarcoma and leukemia viruses (ASLVs) in mammalian and chicken cells. He ingeniuosly exploited the panel of inbred chicken lines maintained at our Institute in Prague. The inbred lines were established in the 1950s and were widely used for Milan Hašek’s experiments on immune tolerance and later for studies on avian immunogenetics. The inbreeding caused segregation of resistant alleles for ASLV receptors, so that different inbred lines displayed either susceptibility or resistance to distinct ASLV subgroups. This appeared to be useful in the subgroup definition and identification of receptor molecules, specifically of the receptor for the C subgroup. Jan was also interested in the neo-antigenic effect of transduced oncogenes and inspired experiments with v-*src* DNA induction of sarcomas in chickens. This turned out to be a fruitful field for further studies on tumor regression, metastasis, and anti-cancer vaccines.

It was quite natural that after the collapse of the communist regime in 1989, Jan became the Director of the Institute of Molecular Genetics. He always openly expressed his opinions on politics, public issues, and management of science. Jan was entirely loyal to the Institute and the Academy of Science as a whole, and he used his scientific authority for the benefit of the research community in Czechia. His directorship during the years 1991–1999 was framed with social turmoil and turbulences, insufficient funding, and painful reform of science. The Czech Academy of Sciences even faced political forces aimed at complete dissolution of basic research or, at least, subverting the Academy to the universities. It cost Jan much strength to resist, but at the end he could hand over the Institute in undiminished form and ready for the next heyday.

**Fig. 2:**
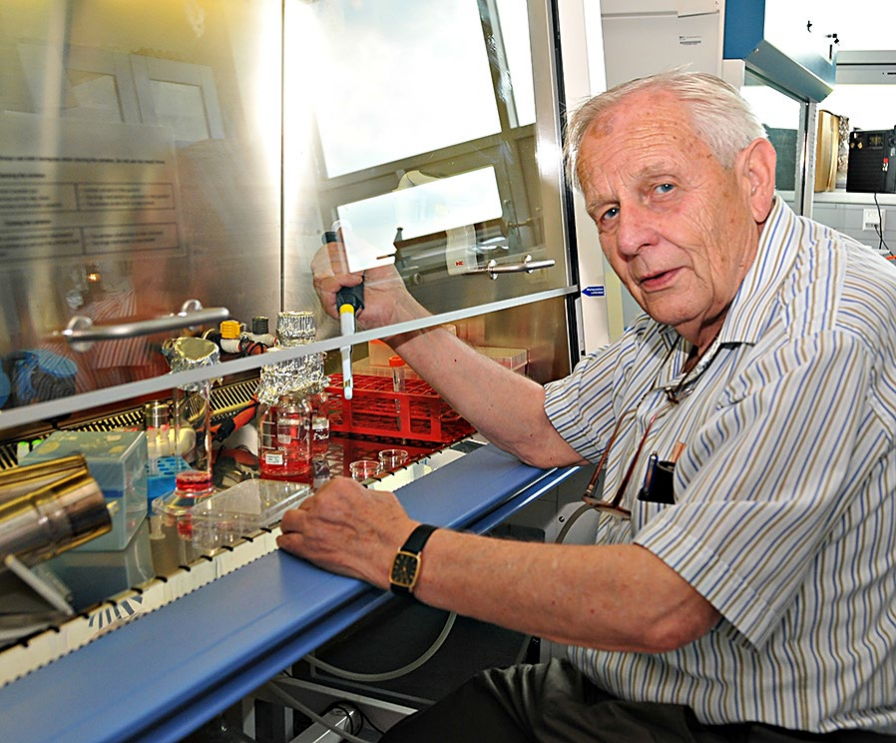
Old love never dies. Jan Svoboda enjoyed cell culture until his last days. The picture was taken in 2014 when he was 80

Over the ensuing years, Jan came back to the problems and questions originating in the late 1950s. At that time, the non-permissiveness of mammalian cells to RSV replication was a happy circumstance in the definition of provirus. But what factors made the chicken cell permissive for RSV? And what factors had to be provided to the mammalian cell for an efficient virus rescue? Jan Svoboda touched many facets of this problem, particularly the virus entry, virus mRNA expression and splicing, and structural refolding of virus envelope glycoproteins during virus-receptor interactions. To do this, Jan came back to the bench (Fig. 2) and learned new methods and instrumental techniques. It was not easy, but Jan’s stubbornness and his nose for experiments guaranteed success. He remained fully active in the laboratory even in the very last years, when his pulmonary problems stealthily cut into his physical capacity and resulted in big discomfort.

Over the course of his career, Jan trained many students and fellows, and he often said that building the scientific school was his most important legacy. He was skilled in identifying talented people and he viewed the training as an intimate collaboration. It was not easy to be Jan’s student or associate, but it stimulated creativity and encouraged independence. Jan’s whole-life scientific activity gained him multiple awards and honors, including national prizes for science Czech Head and Neuron and the membership in EMBO and the Learned Society of the Czech Republic. In 2015, he was elected as Foreign Associate of the National Academy of Science of the USA. Jan himself particularly much appreciated that the scientific community honored him with organization of the Centennial Retrovirus Conference in Prague in 2010.

Jan was complex, brilliant and deeply dedicated to science. He was a philosophical man of eclectic interests and myriad of accomplishments. Jan was also a committed debater, and it used to be delightful to discuss or argue with him not only science, but also politics, history, and philosophy. He was able to cite the ancient classics and often entertained the company with apt quotes from The Good Soldier Švejk, the Czech satirical novel by Jaroslav Hašek.

The field of retrovirology says goodbye to one of its giants. Our condolences go to his wife Ingrid, his sons Jan and Václav, and the rest of his family.

